# MARZ: an algorithm to combinatorially analyze gapped *n*-mer models of transcription factor binding

**DOI:** 10.1186/s12859-014-0446-3

**Published:** 2015-01-31

**Authors:** Rowan G Zellers, Robert A Drewell, Jacqueline M Dresch

**Affiliations:** 1 Department of Computer Science, Harvey Mudd College, 301 Platt Boulevard, Claremont CA, 91711 USA; 2 Department of Mathematics, Harvey Mudd College, 301 Platt Boulevard, Claremont CA, 91711 USA; 3 Biology Department, Clark University, 950 Main Street, Worcester MA, 01610 USA; 4 Department of Mathematics and Statistics, Amherst College, P.O. Box 5000, Amherst MA, 01002 USA

**Keywords:** Transcription factor, Binding site, Position weight matrix, Gene regulation

## Abstract

**Background:**

A key challenge in understanding the molecular mechanisms that control gene regulation is the characterization of the specificity with which transcription factor proteins bind to specific DNA sequences. A number of computational approaches have been developed to examine these interactions, including simple mononucleotide and dinucleotide position weight matrix models.

**Results:**

Here we develop a novel, unbiased computational algorithm, MARZ, that systematically analyzes all possible gapped matrices across a fixed number of nucleotides. In addition, to evaluate the ability of these matrix models to predict *in vivo* binding sites, we utilize a new scoring system and, in combination with established scoring methods and statistical analysis, test the performance of 32 different gapped matrices on the well characterized HUNCHBACK transcription factor in *Drosophila*.

**Conclusions:**

Our results indicate that in many cases gapped matrix models can outperform traditional models, but that the relative strength of the binding sites considered in the analysis can profoundly influence the predictive ability of specific models.

**Electronic supplementary material:**

The online version of this article (doi:10.1186/s12859-014-0446-3) contains supplementary material, which is available to authorized users.

## Background

To understand the biological process of gene expression at the molecular level we must comprehend the nature of the chemical binding events that occur between proteins and DNA. More specifically, in the field of transcriptional regulation, the identification of transcription factor (TF) binding sites is crucial to our understanding of *cis*-regulatory modules and their function in the control of gene regulation.

For over three decades, computational biologists have been working to develop better approaches to predict the binding events that take place between TF proteins and DNA. One of the most widely used approaches, the Position Weight Matrix (PWM) model was introduced in the 1980’s [1-3]. This approach relies on two key assumptions [3]. The first is that DNA sequences that share the same physical binding affinity for a specific TF are equally likely to be present in the genome. The second is that the binding energies for TF contacts with each individual nucleotide in a binding site are additive (i.e., nucleotide positions within the TF binding site are independent of each other).

The approximation of the binding energy of a nucleotide at a particular position within a sequence depends on both the frequency at which that nucleotide is observed in the experimentally determined protein binding sites (recorded in the PWM), and the background frequency corresponding to that nucleotide (i.e., the genome-wide nucleotide distribution) [4]. In many cases, the binding affinity of any particular sequence is calculated relative to the consensus sequence, the sequence constructed from the most commonly found nucleotide at each position in the binding site. These simple PWM models have been effectively implemented and shown to provide reliable approximations for binding of a range of different prokaryotic and eukaryotic TFs [5-8]. However, in more recent biochemical studies, dependencies between neighboring nucleotides in binding sites have been observed and many groups have begun to take such dependencies into account by building more complex models of protein-DNA binding [9-18]. Current experimental methodologies do not allow for the single base-pair or strand-specific resolution of binding sites, emphasizing the need to employ a systematic and non-bias approach to investigate the composition of TF binding sites.

Two general extensions to traditional PWM models are dinucleotide and *n*-mer models. These approaches are implemented in a similar way to the traditional mononucleotide PWM, but weaken the assumption of independence of contiguous nucleotides in the binding site. Dinucleotide models consider dependence between adjacent nucleotides, while *n*-mer models consider a contiguous group of *n* nucleotides, instead of the traditional single nucleotide [13,15,19].

There are now many publicly available algorithms for determining TF-DNA binding specificity. Weirauch et al, in their 2013 publication, systematically compare 26 different algorithms on protein binding microarray (PBM) data from 66 different mouse TFs [20]. The 26 models they analyze include traditional PWM-based models, dinucleotide models, and *n*-mer models. Their results support the idea that, for some TFs, *n*-mer models may perform better overall than simpler models. However, their study also highlights the important roles that the specific experimental data used, as well as the evaluation criteria, play in such a comparison. They state that, although surprising, “the appearance and information content of a motif has little bearing on its accuracy” [20].

Even more recent approaches addressing nucleotide dependence have used a variety of different techniques [16-18]. In these studies, results are shown to illustrate the improvement these different approaches give over traditional PWMs and/or other standard approaches. One may note that each of these recent studies still considers contiguous nucleotide dependence, with some flexibility for gaps between half sites, but none of these studies has systematically looked at different combinations of non-adjacent nucleotide dependence. These conclusions have led us to investigate the role of nucleotide dependence in binding sites and develop a novel and intuitive scoring method for comparing all possible models of nucleotide dependence with no inherent biases.

To better understand nucleotide dependencies in TF binding sites, we begin with a systematic approach aimed at comparing weight matrices produced by each possible gapped *n*-mer across a fixed number of nucleotides. This approach allows for any specific number and arrangement of nucleotides within a sequence to be ignored when considering dependent/independent binding. We have developed a new algorithm, MARZ (combinatorial Matrix Analysis and Ranking inspired by Zero-knowledge proofs), which allows us to investigate all possible gapped *n*-mers of a particular length, test them on *in vivo* TF binding data, and statistically compare their performances with standard mononucleotide-based (PWM) and dinucleotide-based models [1-3,13,17,21].

## Methods

**Figure 1:**
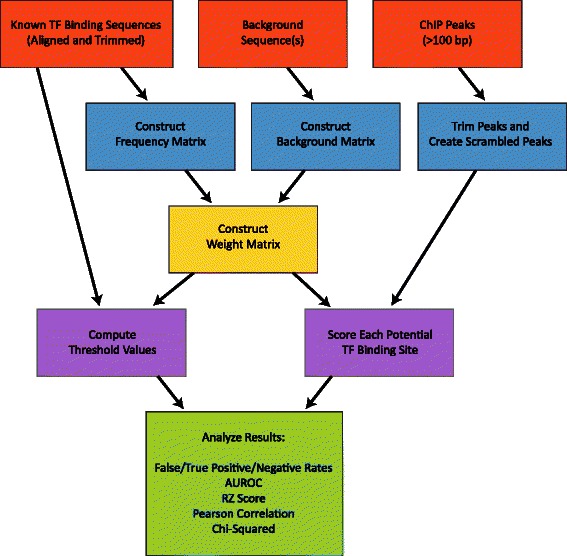
**Complete Flowchart of the MARZ algorithm.** The flowchart illustrates the MARZ algorithm during implementation. The figure should be read from top to bottom, as the three boxes at the top (orange) illustrate the three inputs to the MARZ algorithm and the large box at the bottom (green) illustrates the values the MARZ algorithm outputs.

The complete MARZ algorithm is illustrated in the flow chart shown in Figure 1. What follows is a detailed description of each individual component of the algorithm.

### Gapped *n*-mers

We begin by defining a gapped *n*-mer. Let *k* represent a nucleotide we ignore, and *m* represent a nucleotide we consider. One should first note that we only consider gapped *n*-mers that begin and end with an *m*, thus assuming that the gapped *n*-mer represents a minimal length string of dependent nucleotides that contribute to binding. Allowing a *k* on either end would allow for leading or terminal nucleotides that do not contribute to binding events.

**Figure 2:**
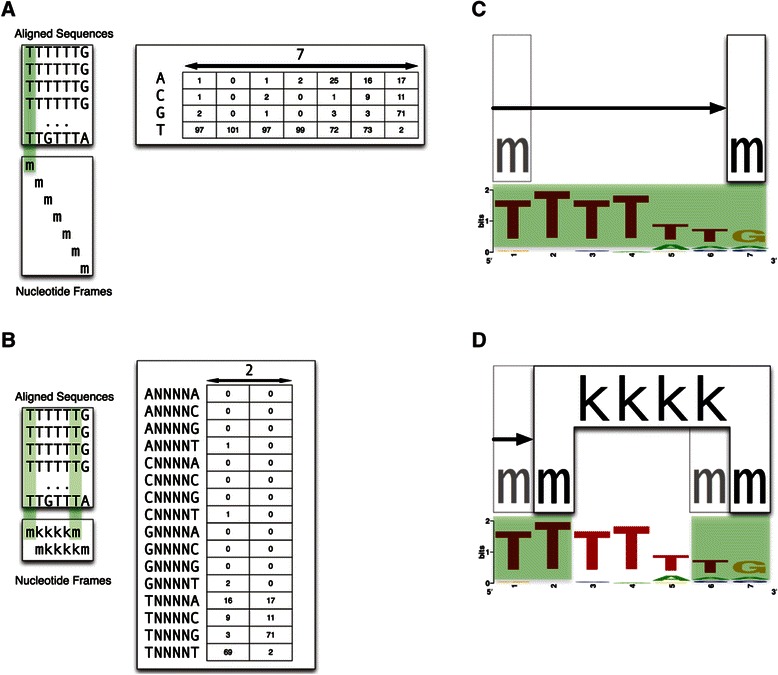
**Comparison of a traditional frequency matrix and a gapped** ***n*** **-mer matrix for HB.** **(A)** The traditional matrix treats each of the seven nucleotides (m) in the aligned HB binding site sequences independently and slides across the sequences with a window frame size equal to one nucleotide. The 4×7 matrix represents the frequency at which each of the four nucleotides is found at each of the seven positions in the binding site and can be used to generate a standard position weight matrix (PWM). **(B)** The matrix for the gapped *n*-mer *mkkkkm* considers each of the two outer nucleotides (m), but ignores the four inner nucleotides (k), with a sliding window frame size equal to six nucleotides. This generates a 16×2 matrix that represents the frequency at which the 16 possible nucleotide pairs are found at the two outer positions across the two possible frames in a seven nucleotide binding site sequence. It should be noted that the complex matrix constructed for each of the 32 different gapped *n*-mers is generated using the same principles as the *mkkkkm* example above, but is distinct based on the specific composition of the gapped *n*-mer. **(C-D)** Corresponding visualizations of each matrix at a HB binding site.

To create a simple numbering scheme for each matrix type and illustrate the non-bias nature of the matrix types included, each gapped *n*-mer has a unique ‘Type ID’ corresponding to the binary encoding of *k*’s and *m*’s. The list of type IDs considered contains every integer from 0 to 31, with the one-to-one correspondence between the type ID and gapped *n*-mer defined as follows:

First, convert the type ID to binary. Then convert the binary representation to a string of *k*’s and *m*’s representing the nucleotides considered in that particular model by replacing each zero with a *k* and each one with an *m*. Any leading *k*’s are omitted, as the leading nucleotide must be included, and an *m* is inserted on the right hand side, as the terminal nucleotide is always included. Table 1 lists all of the type IDs and Figure 2 gives a graphical illustration of the matrix construction and sequence interpretation for the mononucleotide model *m* and the more complex gapped *n*-mer model *mkkkkm*.

**Table 1:** **The first column lists the Type ID for each gapped** ***n*** **-mer, the second column lists the binary representation of the Type ID, the third column lists the k/m representation for each binary representation, obtained by replacing each 0 with a k and each 1 with an m, and the fourth column lists the final corresponding k/m representation of each gapped** ***n*** **-mer, obtained by removing all leading k’s and adding an m to the end of each entry in column three**

**Type ID**	**Binary representation**	**k/m Representation**	**Gapped** ***n*** ** -mer**
0	00000	kkkkk	m
1	00001	kkkkm	mm
2	00010	kkkmk	mkm
3	00011	kkkmm	mmm
4	00100	kkmkk	mkkm
5	00101	kkmkm	mkmm
6	00110	kkmmk	mmkm
7	00111	kkmmm	mmmm
8	01000	kmkkk	mkkkm
9	01001	kmkkm	mkkmm
10	01010	kmkmk	mkmkm
11	01011	kmkmm	mkmmm
12	01100	kmmkk	mmkkm
13	01101	kmmkm	mmkmm
14	01110	kmmmk	mmmkm
15	01111	kmmmm	mmmmm
16	10000	mkkkk	mkkkkm
17	10001	mkkkm	mkkkmm
18	10010	mkkmk	mkkmkm
19	10011	mkkmm	mkkmmm
20	10100	mkmkk	mkmkkm
21	10101	mkmkm	mkmkmm
22	10110	mkmmk	mkmmkm
23	10111	mkmmm	mkmmmm
24	11000	mmkkk	mmkkkm
25	11001	mmkkm	mmkkmm
26	11010	mmkmk	mmkmkm
27	11011	mmkmm	mmkmmm
28	11100	mmmkk	mmmkkm
29	11101	mmmkm	mmmkmm
30	11110	mmmmk	mmmmkm
31	11111	mmmmm	mmmmmm

The fourth column illustrates which nucleotides are considered (m) and which are ignored (k) when scoring a potential binding site.

From this definition, one can easily see that the mononucleotide model *m* has an ID of 0. Likewise, the dinucleotide model *mm* has an ID of 1. For a more complex example, consider the gapped *n*-mer illustrated in Figure 2B. This gapped *n*-mer has ID 16 (Table 1), which can be converted to the binary number 10000. Following the above description, we replace each zero with a *k*, each one with an *m*, and insert an *m* on the right hand side. This results in the gapped *n*-mer represented in Figure 2D, *mkkkkm*. This conversion from a type ID to a gapped *n*-mer, including the intermediate steps, is shown in Table 1 for all 32 matrix types.

### Data required

For each TF, the MARZ algorithm takes as input:

1. A file of aligned sequences (from footprint and/or protein binding microarray experiments), representing known binding sites. Each sequence must be of the same length.
2. A collection of Chromatin Immunoprecipitation (ChIP) peaks.
3. A sequence of DNA that is representative of the background nucleotide composition.

For this study, in an effort to avoid introducing any inherent bias which may be included in short stretches of DNA sequence, we use the sequence from the entire *Drosophila* genome for the background [22]. However, the choice of which DNA sequence is utilized for the background in the MARZ algorithm is at the discretion of the user.

### Constructing a Weight Matrix and Scoring a Sequence

All parameters defined in this section are listed in Table 2, with the value(s) used for each parameter during implementation in the case of the HUNCHBACK TF.

**Table 2:** **List of variables, definition of each variable, and the value(s) used during the implementation of MARZ with respect to HB**

**Variable**	**Definition**	**Setting**
*l*_*n*_	Gapped *n*-mer length	*l*_*n*_≤6
*μ*	Number of nucleotides considered	*μ*≤6
*κ*	Number of nucleotides ignored	*κ*≤4
*l*	Length of each potential HB binding sequence	*l*=7
*L*	Number of aligned HB binding sequences	*L*=101
*N*	Number of HB ChIP peaks	*N*=3142
*l*_*c*_	Length of each HB ChIP peak	*l*_*c*_=100
*P*	Number of scrambles per ChIP peak	*P*=100

#### Creating a weight matrix from binding site data

For construction of weight matrices, we generalize traditional scoring formulas given by Bucher, Hertz and Stormo, and Gershenzon et al. [6,21,23]. A motif is a string of nucleotides with a length *l* corresponding to the length of each aligned sequence. First, define *κ* to be the number of gapped nucleotides, *k*, and *μ* to be the number of nucleotides we consider, *m*, in the gapped *n*-mer. Let *l*_*n*_ be the total number of nucleotides in the scoring frame, meaning *l*_*n*_=*κ*+*μ*. Then, the gapped *n*-mer matrix contains 4^*μ*^ rows (one for each combination of nucleotides at the *μ* positions we are considering), and (*l*−*l*_*n*_+1) columns. Let *n*_*bi*_ be the number of known binding sites that have the gapped *n*-mer *b* starting at position *i* of the motif. Let *e*_*b*_ be the expected proportion of gapped *n*-mers represented by the sequence *b*. This value is calculated from a sequence of DNA that is representative of the background nucleotide composition. Then, the formula for the weight of *b* at position *i* of the motif is given by:

$$\begin{aligned} w_{bi} = \ln \left(\frac{n_{bi}+e_{b}}{e_{b}\left(L+1\right)}\right) + c_{i} \end{aligned} $$

where *L* is the total number of known binding sites and $c_{i} = - \displaystyle \max _{b \in B} \ln \left (\frac {n_{\textit {bi}}+e_{b \in B}}{e_{b \in B}\left (L+1\right)}\right)$, such that the maximum weight in each column is 0.

One should note that we must incorporate pseudocounts, or Dirichlet smoothing, into our calculations to avoid taking the natural logarithm of zero or dividing by zero. We therefore add a pseudocount to *e*_*b*_ in the following way:

Let *B* be defined as the set of all 4^*μ*^ gapped *n*-mers. For all *b*∈*B*,

$${ \fontsize{7.3}{6}\begin{aligned} e_{b} = \cfrac{\left(\text{\# of subsequences matching }b \text{ in the background sequence} \right)+\cfrac{1}{\left|B\right|}}{\left(\text{total \# of subsequences of length }l_{n}\text{ in the background sequence}\right) +1} \end{aligned}} $$

Note that in the above formula |*B*|=4^*μ*^.In the calculation of *w*_*bi*_ above, we have introduced an analogous pseudocount, following the calculation of Hertz and Stormo [6], by including *e*_*b*_ in the numerator and dividing by *L*+1 instead of *L*. In practice, the user has the option of defining their own background probabilities, *e*_*b*_, but should be careful to avoid setting *e*_*b*_=0.

#### Calculating the weight score *S* for a given sequence

The weight score for a sequence *σ* of *l* nucleotides is calculated using the following formula:

$$\begin{aligned} S_{\sigma} = 1 - \frac{\sum\limits_{b\in B} \sum\limits_{i=1}^{l-l_{n}+1} \delta\left(b,\sigma_{i}\right)w_{bi}}{\sum\limits_{i=1}^{l-l_{n}+1} \displaystyle\min_{b\in B}(w_{bi})} \end{aligned} $$

where *σ*_*i*_ is the subsequence of *σ* from *i* to *i*+*l*_*n*_−1 and

$$\begin{aligned} \delta\left(b,\sigma_{i}\right) = \begin{cases} 1 & \text{if the nucleotides of}~ \sigma_{i} \text{ match} ~b \\ 0 & \text{otherwise.} \end{cases} \end{aligned} $$

Recall that we use the *c*_*i*_ term to set the maximum value in each matrix column to 0. Therefore, one can note that *S*_*σ*_∈[ 0,1].

#### Scoring thresholds

For each sequence, *σ*, of adjacent nucleotides in a ChIP peak, if *S*_*σ*_ is greater than or equal to a fixed ‘scoring threshold’, then that sequence is referred to as a binding site.

There are two ways one can set the scoring threshold. First, the user can manually enter in some threshold to be used for each matrix. Second, the user can enter a percentile, forcing the program to dynamically calculate a threshold based on the experimentally obtained aligned sequence data.

We refer to this percentile as a threshold position *x*∈[0,1]. To understand how this relates to a percentile, note that the threshold, *τ*, used when *x*=0.25 corresponds to the highest threshold at which aligned sequences in the 25th percentile of the experimentally obtained sequences would be identified as binding sites by the algorithm.

One may want to compare the performance of a matrix at a variety of thresholds, interpreting the predictions as including only strong binding sites vs. predictions also including weaker binding sites. MARZ thus has an option for running the algorithm over all thresholds corresponding to percentiles from a known set of binding sites.

### Measuring the Effectiveness of MARZ

#### Sensitivity and Specificity

The effectiveness of a given matrix is measured by comparing its false positive and false negative rates with its true positive and true negative rates. The true positive and false positive rates are often referred to, respectively, as the sensitivity and specificity of the algorithm [21]. We define these rates with respect to each individual matrix’s performance.

True ChIP peaks are defined for ChIP-chip data as the middle 100 base pairs of each peak (similar to the definition used in [20]) and referred to as ‘real’ ChIP peaks. Any ChIP peaks that are less than 100 bp in length are excluded from the analysis. False ChIP peaks are defined by ‘scrambled’ ChIP peaks, consisting of those obtained by randomly shuffling each true ChIP peak. *P* scrambled ChIP peaks are generated for each true ChIP peak by applying the C++ function std::random_shuffle to each ChIP peak. This function permutes each of the nucleotides on the ChIP peak, such that each scrambled ChIP peak has the same number of *A*, *C*, *G*, and *T* nucleotides as the true ChIP peak, but in a random order. For this approach, the random seed is set using the system time [24].

We consider binding sites predicted on a true ChIP peak to be true positives, and those found on a scrambled ChIP peak to be false positives. A matrix identifies a DNA sequence (either a real or scrambled ChIP peak) as a positive if it finds any binding sites within that sequence. It identifies it as a negative if it finds no binding sites.

Recall that for each of the *N* real ChIP peaks we consider *P* scrambled representations of the same nucleotides. We thus define the *true positive rate*, *TPR*, and *false positive rate*, *FPR*, as follows:

$$\begin{aligned} TPR &= \frac{TP}{TP + FN} = \frac{TP}{N} \\ FPR &= \frac{FP}{FP + TN} = \frac{FP}{P \cdot N} \end{aligned} $$

where *TP*, *FP*, *TN*, and *FN* correspond to the number of true positives, false positives, true negatives, and false negatives, respectively.

#### AUROC - Area under receiver operating characteristic

The Area under a Receiver Operating Characteristic curve (AUROC) for each matrix type represents the probability that a binding site is found in a randomly chosen true ChIP peak and not found in a randomly chosen scrambled ChIP peak at any given threshold. A Receiver Operator Characteristic (ROC) curve is a plot of the true positive rate vs. the false positive rate of a test over all possible threshold levels.

To compute the area under the curve, we use the trapezoidal rule of numerical integration. Additionally, for plotting the ROC of a given matrix and computing the AUROC, we add the points (0,0) and (1,1) for TFs for which they are not obtained computationally at any threshold, since, in theory, all ROC curve graphs should contain those endpoints [25].

#### An Alternative to AUROC: RZ score

The AUROC method works well for many problems, but has considerable limitations with respect to its application to the MARZ algorithm. These include:

- First, several of the points in the range [0,1]×[0,1] are biologically irrelevant. For example, having *F* *P* *R*>*T* *P* *R* or *T* *P* *R*≈0 are both unacceptable for practical applications. Using the MARZ algorithm, each matrix type can predict binding sites for scoring thresholds greater than 0. However, thresholds in the range [0,1] may not produce the points (0,0) or (1,1), or many points in the neighborhoods of these points. In fact, since the highest threshold position used, *x*=1, still considers the strongest binding sites to be true binding sites, to produce the point (0,0) on a ROC curve it may require that we go beyond this maximum threshold, which was determined from the experimentally obtained binding sequences.
- Second, merely computing the overall AUROC score loses information about the predictive power of the matrix type at a given threshold. The AUROC gives us no information about which matrix would perform best at a given threshold (i.e., one corresponding to only strong binding sites), since it is a statistic derived from the performance of the matrix over all thresholds, not at a specific threshold.

To address the limitations stated above, MARZ uses an alternative scoring method in addition to the AUROC approach. This method is somewhat analogous to the cryptography concept of the zero-knowledge proof. In its simplest form, a zero knowledge proof is one in which one party can verify that another party has access to some piece of information, without learning anything about the content of that piece of information [26]. The main goal of the MARZ algorithm is to determine whether a given matrix can reliably tell apart real ChIP peaks from scrambled ChIP peaks at a given scoring threshold.

Let *P* be the number of scrambled ChIP peaks corresponding to each true ChIP peak. The scoring algorithm considers each true ChIP peak, *C*_*i*_, and its corresponding set $\hat {C}_{i} = \left \{\hat {C}_{i,1}, \ldots \hat {C}_{i,P}\right \}$ of scrambled peaks. First, for each ChIP peak, we consider the number of predicted binding sites in *C*_*i*_ and the average number of predicted binding sites over the set $\hat {C}_{i}$. For a given threshold, *τ*, we define

$$\begin{aligned} r_{C_{i}} &= \left\vert\left\{ \sigma\; \left|\sigma\ \text{is a contiguous substring}\right.\right.\right.\\ &\left.\left.\text{of }C_{i} \text{ of length }l\text{ and}~ S_{\sigma} \ge \tau\right\} \right\vert \\ a_{\hat{C}_{i}} &= \frac{\sum\limits_{j =1}^{P} r_{\hat{C}_{i,j}} }{P}\\ \end{aligned} $$

For any matrix type, given a true ChIP peak and a set of scrambled ChIP peaks, there are three possibilities:

1. MARZ is able to correctly identify the true and scrambled ChIP peaks as such.
2. MARZ incorrectly identifies the true and scrambled ChIP peaks as such.
3. MARZ is unable to identify which ChIP peaks are true and which are scrambled.

The RZ scoring system seeks to reflect which of these possibilities each matrix type most often results in. If the number of predicted binding sites on a true ChIP peak is greater than the average number of predicted binding sites on the corresponding scrambled ChIP peaks, a point is added to the score. If the average number of predicted binding sites on the scrambled ChIP peaks is greater than the number of predicted binding sites on the corresponding true ChIP peak, 0 is added to the score. Otherwise, 0.5 is added to the score. Hence, for each ChIP peak the individual peak’s RZ score is given by:

$$\begin{aligned}</p><p class="noindent">z\left(C_{i},\hat{C}_{i} \right) = \begin{cases} 1 & \text{if}~ r_{C_{i}} - a_{\hat{C}_{i}}>0.5 \\ 0.5 & \text{if}~ -0.5<r_{C_{i}} - a_{\hat{C}_{i}}\leq 0.5 \\ 0 & \text{if}~ r_{C_{i}} - a_{\hat{C}_{i}}\leq -0.5 \end{cases} \end{aligned} $$

Note that 0.5 was chosen in the above formula since $r_{C_{i}} \in \mathbb {Z}$ and $a_{\hat {C}_{i}} \in \mathbb {Q}$.

The overall RZ score for a specific matrix type, TF, and scoring threshold, is then defined as:

$$RZ = \frac{\displaystyle\sum_{i = 1}^{N} z\left(C_{i},\hat{C}_{i} \right)}{N} $$

#### The RZ score of a random guesser

One key advantage of the AUROC method is that there is a natural baseline score to compare results to. An AUROC of less than or equal to 0.5 implies that the matrix type in question has no more predictive power than guessing randomly whether a given sequence represents a binding site or not. The RZ scoring method functions similarly.

For clarity, we define a random guesser as a ‘matrix type’ that predicts a binding site with probability 0.5 at each possible position (using a sliding window of length *l*) along a ChIP peak. This probability is referred to as the discovery rate. One can easily show that the expected RZ score for such a random guesser is 0.5.

#### Comparison to Transcription Factor Flexible Models

To compare the gapped *n*-mer models to previously published models that address nucleotide dependencies, we create both First-order and Detailed Transcription Factor Flexible Models (TFFMs) using the Hidden Markov Model-based algorithm developed by and available from the Wasserman Lab [18]. These are created using the same known binding sites used to construct the gapped *n*-mer models. RZ scores are computed from the predictions found using these TFFM models at 100 different TFFM hit probability/score thresholds (chosen uniformly from 0.01 to 1.0) on the same set of ChIP peaks used to compute the RZ score for the gapped *n*-mer models. The results for HB, using the known binding sites from Ho et al. and the HB ChIP data from MacArthur et al., are shown in the (Additional file 1: Figure S1) [27,28].

#### Statistical significance using the RZ scoring system

For a given TF, we use the Chi-square goodness of fit test to compare the results of a matrix corresponding to a specific gapped *n*-mer to that of the commonly implemented mononucleotide matrix, *m*.

For each matrix type and threshold, we perform a Chi-square goodness of fit test using the number of ‘hits’ (ChIP peaks resulting in $z\left (C_{i},\hat {C}_{i}\right) = 1$), ‘misses’ (ChIP peaks resulting in $z\left (C_{i},\hat {C}_{i}\right) = 0$), and ‘borderlines’ (ChIP peaks resulting in $z\left (C_{i},\hat {C}_{i}\right) = 0.5$) obtained by the MARZ algorithm.

For each Chi-square test, the null hypothesis is that the matrix type being analyzed gives the same results as the mononucleotide matrix, *m*. We compute the Chi-square value as follows:

$$\begin{aligned} \chi^{2} &= \sum_{i=1}^{n} {\frac{(O_{i} - E_{i})}{E_{i}}^{2}} \end{aligned} $$

where *n*=3, *E*_*i*_ represents the total number of hits, borderlines, and misses in the case of the mononucleotide matrix, *m*, and *O*_*i*_ represents the total number of hits, borderlines, and misses in the case of the matrix type being analyzed.

#### Pearson correlation coefficient

One additional feature of the MARZ algorithm is its ability to compute how related any two matrix types are in terms of their predictions for a given transcription factor and threshold value (or position), over *N* ChIP peaks, with a given ChIP peak having length *l*_*c*_.

We create a vector of predictions for each matrix by considering each binding nucleotide separately. A vector of length *l*_*c*_ is created for each ChIP peak. Each element in the vector *v*_*i*_ is set equal to the number of distinct binding sites containing the nucleotide located at position *i*. We then concatenate the vectors for each ChIP peak, creating one vector of length *N*·*l*_*c*_.

After these vectors are constructed for each matrix, the correlation between matrices *x* and *y* is computed using their corresponding vectors, *X* and *Y*. This is done using a slightly modified Pearson correlation coefficient, as described in Section one of the Additional file 1.

### Hierarchical clustering

We use the Pearson correlation coefficient and agglomerative hierarchical clustering to build a tree representing how related the predictions obtained from different matrix types are. The details and results are included in Section two of the Additional file 1.

### Cross-validation

We perform cross-validation with respect to the RZ score, using 50% of the ChIP peaks. The details and results are included in Section three of the Additional file 1.

### Software

Additional file 2, marzscaled.zip, contains a scaled version of the MARZ program. Instructions are in the file ‘runningmarz.pdf’. For a complete version of the MARZ program, contact Jacqueline Dresch.

## Results and discussion

### Application: HUNCHBACK

In order to directly test the performance of the new MARZ algorithm we analyze binding site predictions for the extensively characterized HUNCHBACK (HB) TF. *hunchback* (*hb*) is the primary gap gene of the segmentation regulatory cascade in *Drosophila* [29] and is responsible for establishing the patterning of the anterio-posterior axis in the early embryo [30]. It encodes for a C2H2 zinc finger TF that directly regulates expression of other functionally important gap genes, including *giant* (*gt*), *knirps* (*kni*) and *Kruppel* (*Kr*) [31,32], and pair-rule genes, including *even-skipped* (*eve*) [33]. The relatively simple consensus binding site sequence for HB (TTTTTTG) [27] would seem to present a stringent test of the predictive ability of the different MARZ matrices. To address both the sensitivity and specificity of the MARZ algorithm, we compare the ability of the different matrices to predict binding sites in regions of the *Drosophila* genome shown to recruit HB *in vivo* in ChIP experiments [28].

### Inputs to MARZ

When implementing the MARZ algorithm on HB, we use the following inputs:

1. A file of aligned HB binding sequences [27].
2. A collection of HB ChIP peaks, each of length greater than or equal to 100 bp [28].
3. The entire *Drosophila melanogaster* genome for the background nucleotide composition [22].

The parameters used for the implementation described in this section are listed in Table 2.

### Gapped *n*-mers

The MARZ algorithm utilizes an unbiased, systematically constructed set of 32 matrices (Table 1) to analyze TF binding sequences. The simplest matrix, *m*, is generated from a traditional mononucleotide model in which each nucleotide is considered independently (Figure 2A). When applied to the HB binding sequence, which is seven nucleotides long, this creates seven frames (Figure 2C). A dinucleotide model, *mm*, considers two adjacent nucleotides and an *n*-mer model considers *n* contiguous nucleotides in each frame. In addition to implementing these simple models, our approach examines all possible gapped *n*-mers with up to a six nucleotide frame size. A maximum nucleotide frame size of six was chosen simply to allow for easy visualization of all gapped *n*-mers (Note: a maximum size of seven would result in 64 gapped *n*-mers). When scoring a potential binding site, the gapped *n*-mer matrices only consider a subset of nucleotides (*m*) across any given frame and ignore the other nucleotides (*k*). For example, the *mkkkkm* matrix considers only the two outer nucleotides in each frame. Since the HB binding sequence is seven nucleotides long, using this matrix results in exactly two frames of six nucleotides each (Figure 2B and D).

### AUROC

**Figure 3:**
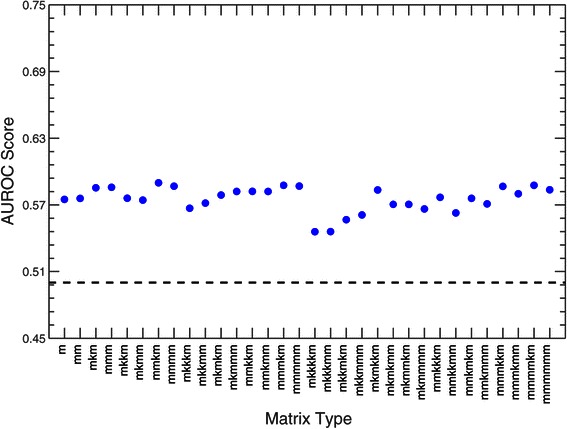
**AUROC score evaluation for HB.** AUROC scores for all 32 gapped *n*-mer matrices for HB. The x-axis corresponds to the gapped *n*-mer used in the MARZ algorithm. The y-axis corresponds to the AUROC score obtained from each gapped *n*-mer’s ROC curve. The dashed line at 0.5 represents the score obtained in the case of no-discrimination. All values fall in the range [0.55,0.59].

We measure the performance of the 32 matrices in predicting HB binding sites by calculating AUROC scores. This analysis consistently produces values in the range [0.55,0.59] (Figure 3). Unfortunately, because of the low sensitivity of AUROC with respect to scoring different matrix types, it is difficult to use this measure to compare the performance of the different matrices.

### RZ Score

**Figure 4:**
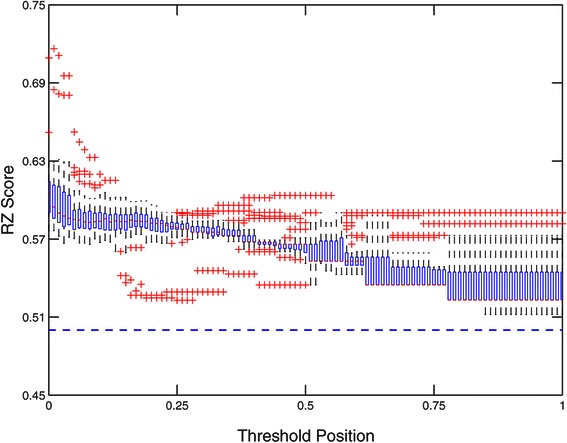
**RZ score evaluation for all 32 gapped** ***n*** **-mer matrices for HB.** The x-axis corresponds to the threshold position used for each run of the MARZ algorithm. The y-axis corresponds to the RZ score obtained from each run. At a given threshold, the central mark represents the median RZ score of all gapped *n*-mer matrices, the boxes enclose the 25th to 75th percentiles of the data set, whiskers extend to all other points not considered outliers, and outliers are plotted separately (red crosses).

The relatively uninformative scores obtained using AUROC led us to investigate alternative scoring methods to assess the performance of the 32 matrices. The RZ score effectively measures the predictive ability of a particular matrix to discriminate between *in vivo* confirmed ‘real’ binding sites contained in ChIP peaks from the genome and sequences from ‘scrambled’ ChIP peaks. In the case of HB (Figure 4), some general trends are observed: i) All 32 matrices outperform a random guesser at all thresholds (all RZ scores above 0.52 are significantly greater, at a significance level of 0.01, than those obtained using the random shuffle). ii) The matrix scores tend to show a monotonic decrease in average score as the threshold position is increased. iii) Performance at any given threshold varies with matrix type. iv) Performance of each matrix changes in response to the threshold used. v) Variation in the performance of different matrix types is greater at extreme thresholds (i.e., those close to 0 and 1).

**Figure 5:**
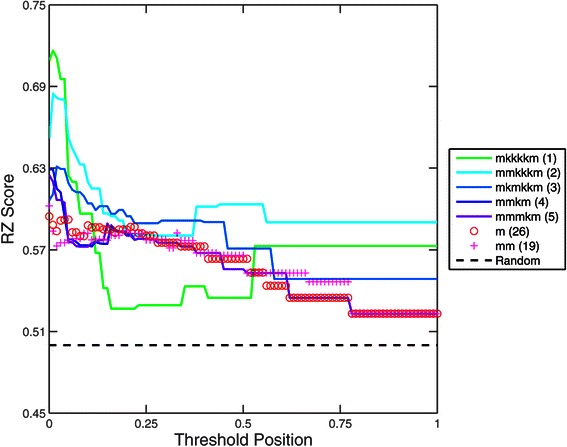
**Performance of top five gapped** ***n*** **-mer matrices for HB across all threshold values.** The x-axis corresponds to the threshold position used for each run of the MARZ algorithm. The y-axis corresponds to the RZ score obtained from each run. Of the 32 matrices, the five with the highest RZ score, along with the mononucleotide (*m*) and dinucleotide (*m* *m*) matrices for comparison, are shown. The ranking of each matrix is indicated in parentheses. All of these matrices consistently outperform the 0.5 RZ score for a matrix that fails to discriminate between true and false positive binding sites (random, dotted line), but do not perform evenly across the different threshold values.

If we consider only the top five ranked matrices by highest peak RZ score (obtained at any threshold value), then it becomes clear that the threshold position is critically important to performance (Figure 5). Each of these top five matrices performs variably across the range of thresholds. For example, the top ranked *mkkkkm* matrix outperforms all other matrices at the 0.01 threshold position, but underperforms almost all other matrices, including the 26^*th*^-ranked *m* and 19^*th*^-ranked *mm*, at the 0.25 threshold (Figure 5).

**Figure 6:**
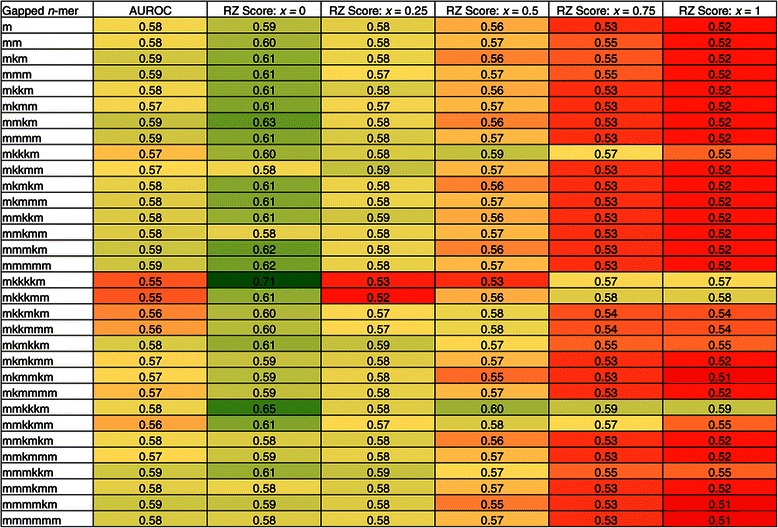
**AUROC and RZ score evaluation for all 32 gapped** ***n*** **-mer matrices for HB.** This heatmap summarizes the results shown in Figures 3 and 4. The first column lists each of the 32 gapped *n*-mers. The second column contains the AUROC score obtained from each gapped *n*-mer’s ROC curve. The third through seventh columns contain the RZ scores obtained from each gapped *n*-mer at the threshold positions 0.0, 0.25, 0.5, 0.75, and 1.0, respectively. For columns two through seven, the scores are color-coded with green, yellow, and red for high, medium, and low values respectively.

A summary of the AUROC and RZ scores are shown in Figure 6. This figure again emphasizes the fact that many of the different gapped *n*-mers are outperforming the traditional mono- and dinucleotide matrices, and that this performance is highly dependent on the threshold position used.

When compared to the RZ scores obtained from TFFMs using the Hidden Markov Model-based algorithm developed by the Wasserman Lab at 100 different TFFM hit probability/score thresholds, the RZ scores obtained from the gapped *n*-mer models perform similarly at many thresholds (Figure 4 vs. Additional file 1: Figure S1) [18]. However, the highest scores obtained from the best performing gapped *n*-mers are higher than those obtained from the TFFMs (Figure 5 vs. Additional file 1: Figure S1). One should note that the best performing gapped *n*-mer results in an RZ score of 0.71, while the TFFMs result in RZ scores below 0.66 at all hit probability/score thresholds.

### Statistical comparison of matrix types

**Figure 7:**
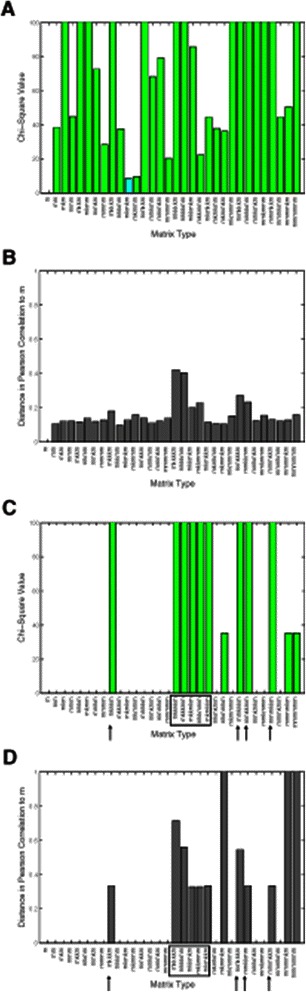
**Comparison of the performance of all gapped** ***n*** **-mer matrices to the traditional** ***m*** **matrix for HB.** **(A and C)** Chi-square values with significance color-coded: Green (*p*<0.01), Aqua (*p*<0.05). See Materials and Methods for a description of the Chi-square analysis. **(B and D)** Pearson correlation distance from mononucleotide matrix. See Materials and Methods for a description of the calculation. Panels **A** and **B** are obtained using a threshold of 0.0, and **C** and **D** using a threshold of 1.0.

To quantify the significance of the performance difference between each matrix and the traditional mononucleotide matrix *m*, we analyze Chi-square and Pearson correlation coefficient values (Figure 7 and Additional file 1: Figure S2). For the Chi-square analysis, we consider how frequently a particular matrix can identify a predicted HB binding site in a ‘real’ ChIP peak relative to ‘scrambled’ ChIP peaks (see Materials and Methods for details). This analysis does not account for whether the results are obtained on the same individual ChIP peaks. To address this issue, we also calculate the Pearson correlation coefficient to investigate at single nucleotide resolution the correlation of the predicted binding sites within each ChIP peak relative to binding sites predicted using the mononucleotide matrix *m*.

For both statistical comparisons, a number of key general trends are observed: i) At the 0 threshold position, all 31 multinucleotide matrices are significantly different from the *m* matrix (Figure 7A), with correlation coefficients less than 0.9 (distance >0.1, Figure 7B). ii) As the threshold position is incrementally increased, fewer matrices remain significantly different from *m*, corresponding to an observed decrease in the correlation coefficients for these matrices (Figure 7B and D, and Additional file 1: Figure S2). iii) A cluster of matrices, including *mkkkkm*, *mkkkmm*, *mkkmkm*, *mkkmmm* and *mkmkkm* (Figure 7C and D, boxed), remain significantly different from *m* with low correlation coefficients (high distances) across the entire range of thresholds. iv) A subset of individual matrices, including *mkkkm*, *mmkkkm*, *mmkkmm* and *mmmkkm* (Figure 7C and D, arrows), are also significantly different from *m* with low correlation coefficients (high distances) across the entire range of thresholds. It should be noted that three of these matrices (*mkkkkm*, *mmkkkm* and *mkmkkm*) are the top three as measured by the highest peak RZ score (Figure 5).

## Conclusions

There are several key conclusions drawn from our implementation of the MARZ algorithm. First, we see that an unbiased and systematic analysis of the predictions from all 32 matrices in the algorithm, including the traditional mononucleotide, dinucleotide and *n*-mer models, and the novel gapped *n*-mer models we developed in this study, is critical to identifying the most robust matrix models. In the case of the HB TF, the performance of many of the gapped *n*-mer models differs significantly from their *n*-mer counterparts. Second, we see that the threshold position at which the analysis is conducted (i.e., the relative strength of the *in vivo* binding sites included in the algorithm, see Figure 1) can profoundly impact the performance of the different matrix models (Figure 4). For example, the gapped *mkkkkm* matrix outperforms all non-gapped *n*-mer models at the 0.01 threshold position (which considers 99% of the known HB binding sites), but does not perform as well at higher thresholds (Figure 5). This observation emphasizes the need for careful consideration of the threshold position in experimental design when investigating TF-DNA binding interactions. A strength of the MARZ algorithm is that it integrates analysis of the predictions of all 32 matrix models across all thresholds for any given TF.

The significant variation in the performance of the 32 matrix models across different threshold positions (Figure 7) highlights the need for rigorous performance assessment methods. In this study, we develop the RZ score to address this goal, in addition to applying existing scoring mechanisms such as AUROC. The RZ scoring method allows for the simple analysis of each of the matrix models at each threshold independently. This approach facilitates the rapid identification of the best performing matrix model(s) and threshold(s) in any given experimental application.

Previous studies on the binding sites for *Drosophila* TFs have indicated that the flanking sequences around identified binding sites may also be important for TF-DNA interactions [34,35]. Using flanking genomic sequences to extend experimentally identified footprints that do not appear to contain a hit to the existing PWM can reveal an extended binding site motif [34]. For many *Drosophila* TFs, including HB, the number of such cases is small (5-10%). In the case of HB, the extension of the consensus motif does not alter the core 7bp binding site, but is achieved through the addition of two neighboring nucleotides (TG), resulting in an extended 9bp motif (TTTTTT(A/G)TG) [34] (http://autosome.ru/DMMPMM/). Application of this extended HB PWM provides increased predictive ability for *in vivo* binding sites when compared to the core 7bp PWM [34,35].

Given the intrinsic difficulty in reliably identifying HB binding sites it will be critical to also consider parallel bioinformatic approaches. Of particular interest will be the clustering of HB sites in the genome [36]. The HB protein has two groups of C2H2-type zinc finger DNA binding domains, separated by over 350 amino acids. One model is that the two groups of zinc-fingers may be capable of contacting distinct binding sites in a stereotypical manner [35]. The topology of these TF-DNA interactions may determine the spatial distribution of the binding sites and therefore it may be important to search for groups of properly spaced and oriented binding sites.

Here we have analyzed a single TF protein, HB. An interesting observation regarding this particular TF is that sequences found to bind HB experimentally all contain a string of T’s. Thus, predictive models often find HB binding to score as well as sites that are offset by a single basepair. This is highlighted by the fact that the best performing gapped *n*-mer at low thresholds, *mkkkkm*, has a string of gapped nucleotides, thus potentially allowing for some wiggle room when binding HB. In the future, it will be very interesting to run a similar analysis on TFs with more information-rich binding sites with less flexibility in their recognition sequences.

A potential limitation for the wider application of the MARZ algorithm to analyze additional TFs is the current lack of availability of either known defined binding sites or genome-wide binding locations from ChIP studies. However, as the cost and technical challenges of such studies diminish in the genomic-era, the availability of these datasets will increase in the coming years. In such cases, the MARZ algorithm will provide a systematic approach to analyze the performance of different matrix models on predicting TF-DNA interactions. As such, it will be critically important to investigate whether the predictive patterns observed for HB-DNA binding with the MARZ algorithm are a common biological feature, by expanding the analysis to include additional TFs in future studies.

## Availability of supporting data

The data set supporting the results of this article is included within the article (and its additional files).

## Additional files

Additional file 1
**Supporting Information.**

Additional file 2
**marzscaled.zip contains a scaled version of the MARZ program.** Instructions are in the file ‘runningmarz.pdf’.
