# Supplementary material for: MARZ: an algorithm to combinatorially analyze gapped n-mer models of transcription factor binding
Source: BMC Bioinformatics. 2015 Jan 31;16:30. doi: 10.1186/s12859-014-0446-3 (PMC4384306; doi:10.1186/s12859-014-0446-3)
Supplement: Additional file 1 — Supporting Information. [file 12859_2014_446_MOESM1_ESM.pdf]

Supporting Information
MARZ: an algorithm to combinatorially analyze gapped n-mer models of transcription factor binding

Rowan G. Zellers [1,2], Robert A. Drewell [3,4,5] and Jacqueline M. Dresch [6,*]

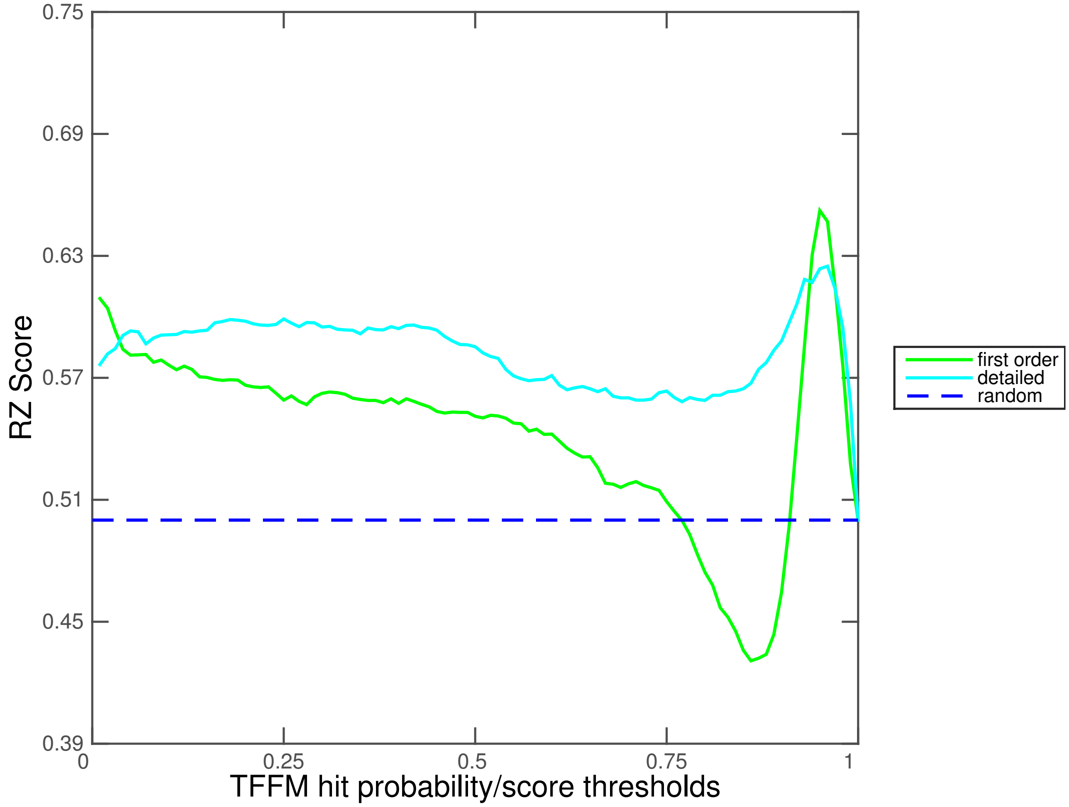

Figure S1: **RZ Scores using the TFFMs for HB.** The x-axis corresponds to the TFFM hit probability / score threshold used for each run. The y-axis corresponds to the RZ score obtained from each run. The RZ scores are highly dependent on the hit probability/score threshold used. More importantly, the highest scores obtained from the TFFMs are lower than those obtained from the best performing gapped n-mer (0.71).

1 Pearson Correlation Coefficient

The standard Pearson Correlation Coefficient is defined as [1]:

$$r(x,y) = \frac{\sum_{i=1}^{n}(X_i - \bar{X})(Y_i - \bar{Y})}{\sqrt{\sum_{i=1}^{n}(X_i - \bar{X})^2}\sqrt{\sum_{i=1}^{n}(Y_i - \bar{Y})^2}}$$

where vectors X and Y correspond to matrices x and y.

We use a modified Pearson correlation coefficient only when the coefficient is undefined (i.e., if $X_i = \bar{X}$ or $Y_i = \bar{Y}$ for all i). In this case, we modify the definition of the Pearson correlation coefficient, r, as follows:

$$r(x,y) = \begin{cases} 1 & \text{if for all } i, X_i = \bar{X} > 0 \text{ and } Y_i = \bar{Y} > 0 \text{ or } X_i = \bar{X} = 0 = Y_i = \bar{Y} \\ -1 & \text{if for all } i, X_i = 0 \text{ and } Y_i = \bar{Y} > 0 \text{ or vice versa} \\ 0 & \text{if for all } i, X_i = \bar{X} \text{ and there exists an } i \text{ such that } Y_i \neq \bar{Y} \text{ or vice versa} \end{cases}$$

This modification guarantees that:

(a) two matrices resulting in a constant number of positive predictions at every nucleotide are defined to have an $r = 1$,

(b) two matrices resulting in no binding site predictions on any of the ChIP peaks are defined to have an $r = 1$.

(c) two matrices such that one predicts no binding sites in any of the ChIP peaks while the other results in a constant number of positive predictions at every nucleotide (ie: opposite predictions) are defined to have an $r = -1$, and

(d) two matrices such that one results in a constant number of positive predictions at every nucleotide while the other does not are defined to have an $r = 0$.

2 Hierarchical clustering

Hierarchical clustering is a method aimed at partitioning objects into groups (clusters) based on their relatedness [2]. This is done by constructing a rooted tree, analogous to phylogenetic trees in evolutionary biology [2]. Here, we use agglomerative hierarchical clustering to build a tree representing how related the predictions obtained from different matrix types are.

2.1 Specifics

There are several algorithms that perform hierarchical clustering. In general, strategies for hierarchical clustering are either agglomerative (bottom up) or divisive (top down) [2]. In agglomerative clustering, one begins with as many clusters as objects, and moves up the hierarchy by joining together clusters. In divisive clustering, one begins with one single cluster containing all objects, and splits clusters recursively.

We use agglomerative clustering with an exhaustive search, as its computational complexity is $O(n^3)$ as opposed to $O(2^n)$ for divisive clustering [2]. Basic pseudocode for the exhaustive agglomerative clustering algorithm is as follows.

```
while numclusters != 1
    imax = 1;
    jmax = 1;
    dmin = inf;
    for i = 1 : numclusters
        for j = i+1 : numclusters
            if dist(i,j) < dmin
                imax = i
                jmax = j
                dmin = dist(i,j)
    cluster(imax,jmax)
    --numclusters;
```

Computing the above pseudocode requires one to calculate the distance between clusters [3]. There are many approaches for calculating this distance[2]. We calculate the distance between clusters by finding the minimum pairwise distance between two potential clades:

$$min\{d(a,b) : a \in A, b \in B\}$$

2.2 Implementation

To investigate the overall relationships between the predictions obtained using the 32 matrices, we calculate the pairwise Pearson correlation coefficients and distances (1− Pearson correlation coefficient) across five different threshold values (Figure S3). In general, at the lower thresholds (Figure S3A and B) the matrices exhibit a lower degree of similarity in their ability to predict binding sites than at higher threshold values (Figure S3C-E). In addition, the top two ranked matrices by highest RZ score (*mkkkkm* and *mmkkkm*) cluster together on the dendrograms irrespective of the threshold value (Figure S3, arrows).

3 Cross validation

To test the model's robustness, and investigate how much the input ChIP peaks impact the ranking of the 32 matrix types, we conduct a cross-validation analysis. We run all 32 matrix types on 10 randomly selected subsets (50%) of the ChIP peaks and compare the top 5 performing matrix types found when the 32 models were tested on the 50% selected versus the 50% non-selected. When this was done five times, little variation in the top performing matrices was observed, with the top two performing matrices remaining the top two in all runs (Figures S4 and S5). This illustrates the robustness of the MARZ algorithm.

References

[1] Rodgers, J. L. and Nicewander, W. A. (1988) *The American Statistician* **42(1)**, 59–66.

[2] Manning, C. D., Raghavan, P., and Schaztze, H. (2008) Introduction to Information Retrieval, Cambridge University Press, New York.

[3] Szekely, G. J. and Rizzo, M. L. (2005) *Journal of Classification* **22(2)**, 151–183.

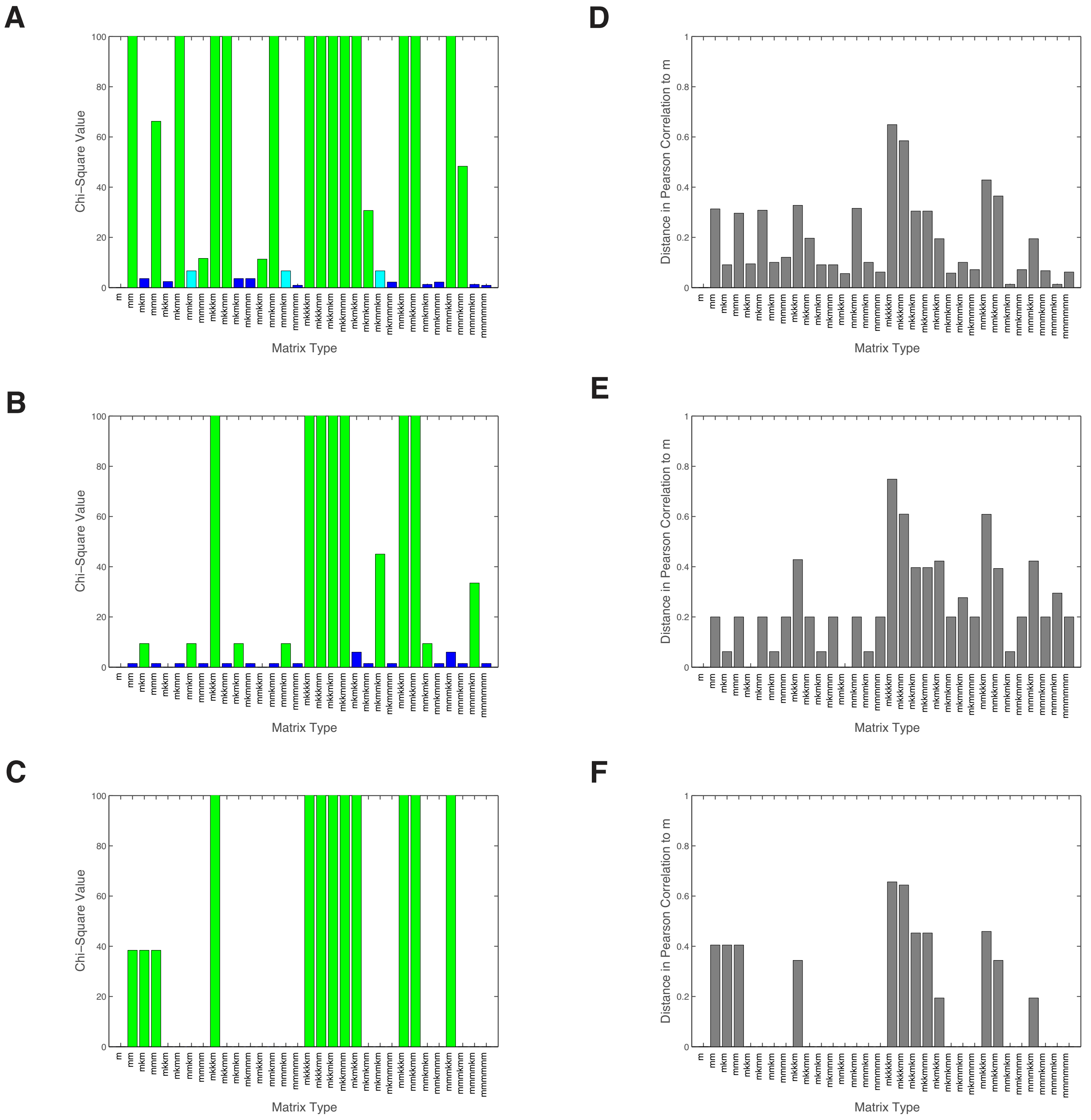

Figure S2: **Comparison of the performance of all gapped n-mer matrices to the traditional m matrix for HB. (A-C)** Chi-square values with significance color-coded: Green ($p < 0.01$), Aqua ($p < 0.05$), Blue ($p \geq 0.05$). See Materials and Methods for a description of the Chi-square analysis. **(D-F)** Pearson correlation distance from mononucleotide matrix. See Materials and Methods for a description of the calculation. Panels **A** and **D** are obtained using a threshold of 0.25, **B** and **E** using a threshold of 0.5, and **C** and **F** using a threshold of 0.75.

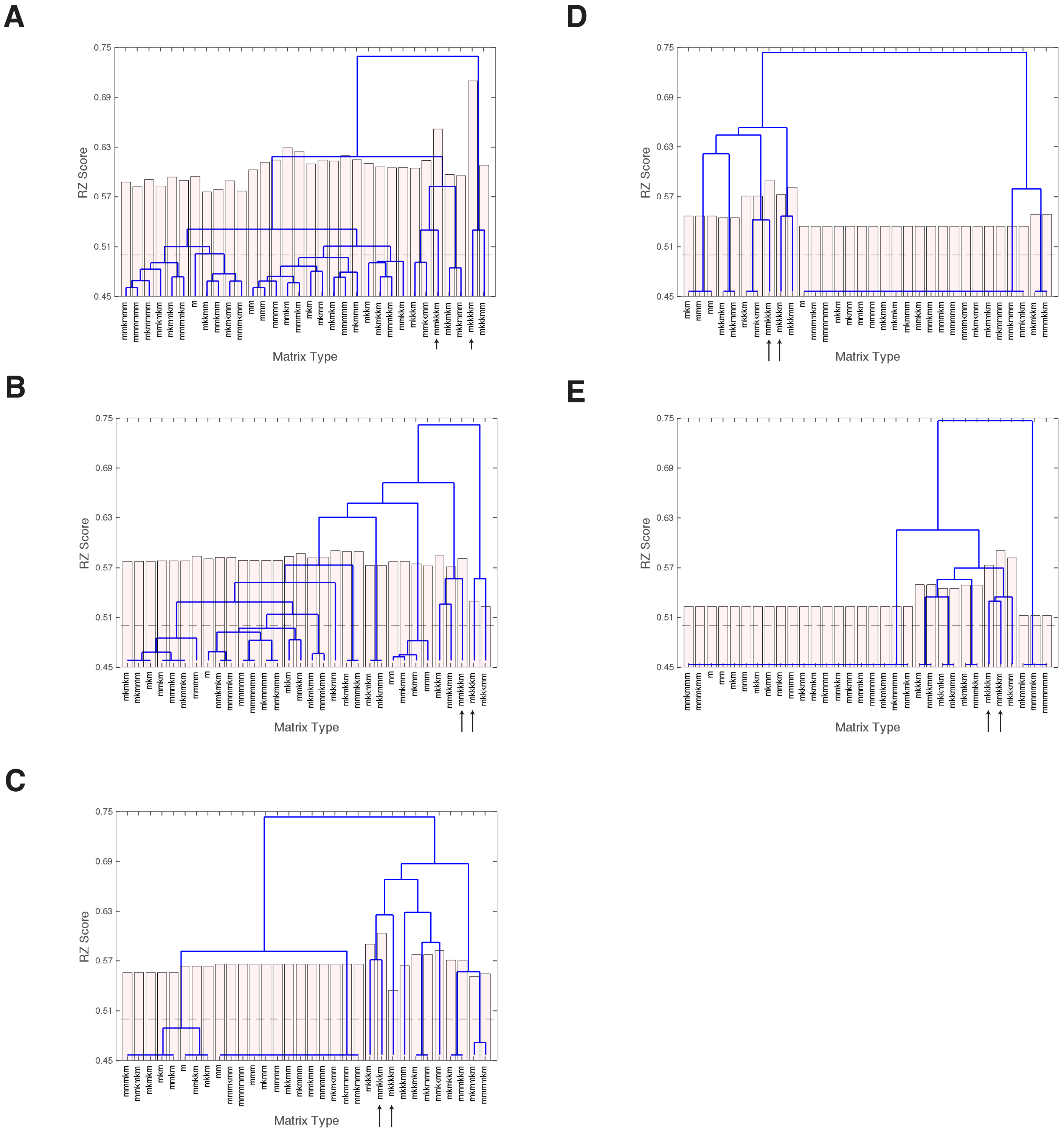

Figure S3: **Matrix type dendrograms for HB.** Dendrograms illustrate the pairwise distance between matrix types, using (1− Pearson correlation coefficient) as the distance between two matrix types. The RZ score is shown in the bar graphs in which the dendrogram is overlaid. Panel **A** is obtained using a threshold of 0.0, **B** using a threshold of 0.25, **C** using a threshold of 0.5, **D** using a threshold of 0.75, and **E** using a threshold of 1.0.

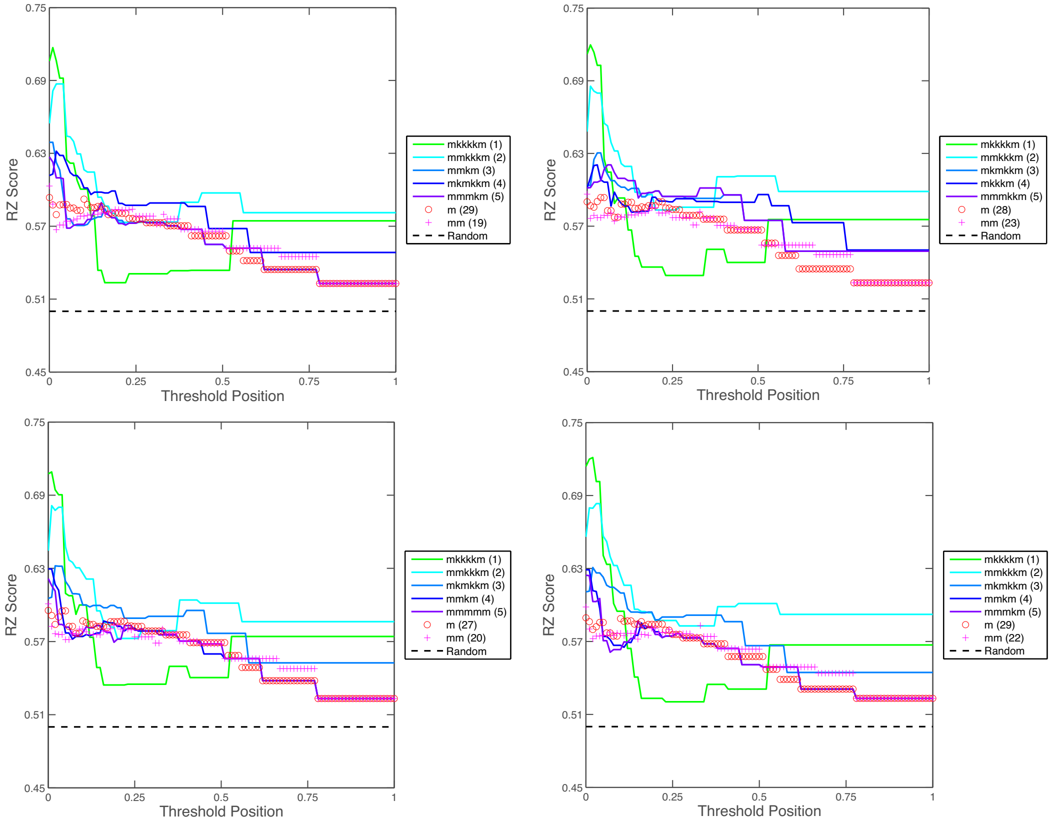

Figure S4: **Cross-validation using 50% of the ChIP peaks for HB.** In each figure, the x-axis corresponds to the threshold position used for each run of the MARZ algorithms. The y-axis corresponds to the RZ score obtained from each run. Of the 32 matrices, the five with the highest RZ score, along with the mononucleotide (m) and dinucleotide (mm) matrices for comparison, are shown. The ranking of each matrix is indicated in parentheses. The 0.5 RZ score corresponds to a matrix that fails to discriminate between true and false positive binding sites (random, dotted line). Figures in the left column represent the performance of the matrices on the randomly selected subsets (50%) of the ChIP peaks. Figures in the right column represent the corresponding performance of the matrices on the 50% of ChIP peaks not selected. The images are continued in Figure S5

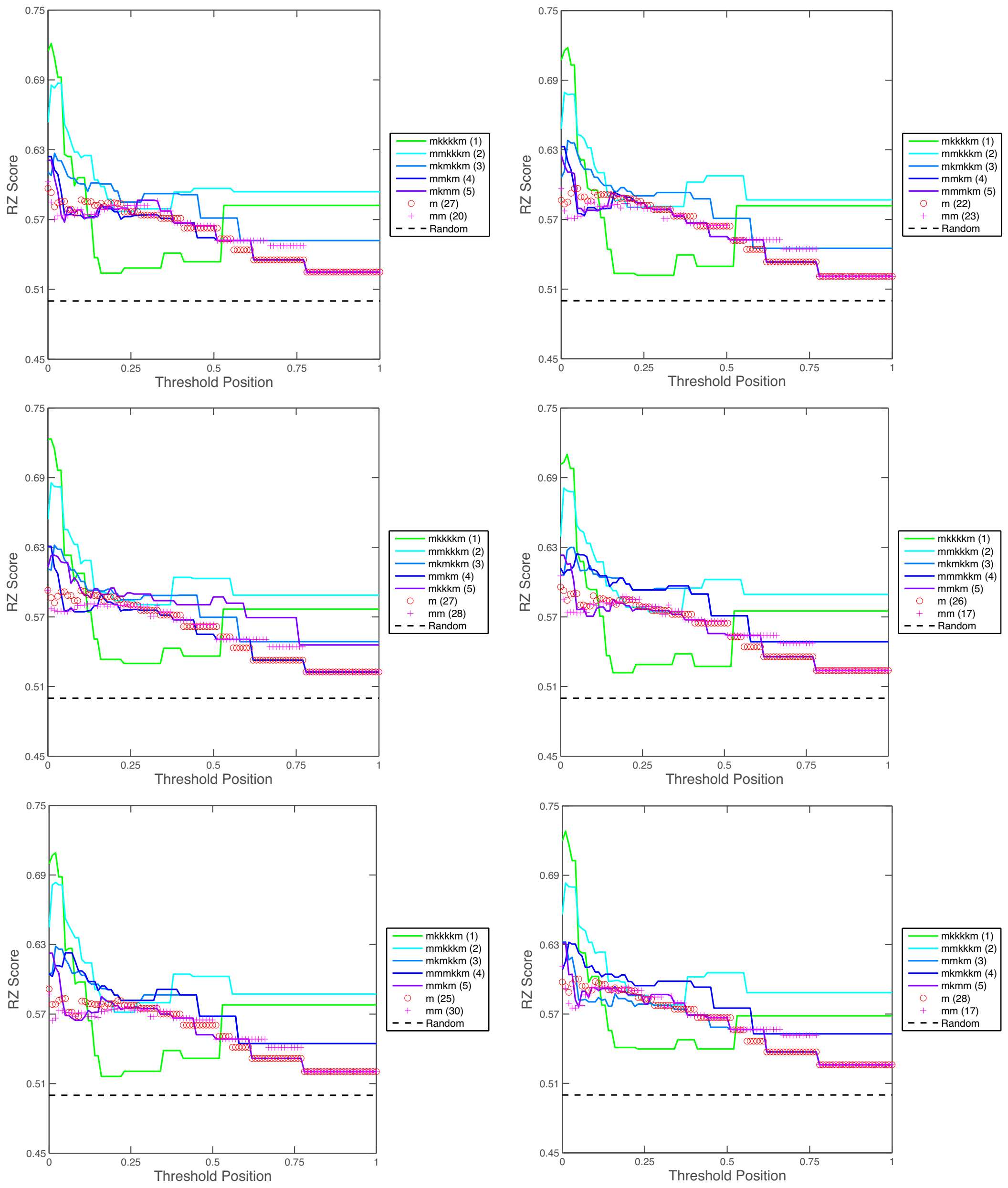

Figure S5: **Cross-validation using 50% of the ChIP peaks for HB continued.**
