# Supplementary material for: MARZ: an algorithm to combinatorially analyze gapped n-mer models of transcription factor binding
Source: BMC Bioinformatics. 2015 Jan 31;16:30. doi: 10.1186/s12859-014-0446-3 (PMC4384306; doi:10.1186/s12859-014-0446-3)
Supplement: Additional file 2 — marzscaled.zip contains a scaled version of the MARZ program. Instructions are in the file ‘runningmarz.pdf’. [file 12859_2014_446_MOESM2_ESM.pdf › marzscaled/runningmarz.pdf]

1 Features of the scaled version of MARZ

The MARZ algorithm described in the paper takes a very long time to run, even on a cluster with many cores. This is because of the combinatorial nature of constructing all possible gapped n-mers and testing them with respect to all possible thresholds. Therefore, the version provided for review is scaled down such that it can be ran (in about 5 minutes) on a laptop.

Unfortunately, this means that several features that the full version of MARZ has are not provided within this version. These are:

(a) Pearson correlation

(b) Running MARZ over all thresholds for a given TF by default

Instead, the MARZ algorithm provided will compute the false and true positives, as well as the RZ score for a given matrix with respect to a given threshold position and a given set of ChIP peaks and aligned sequences. It will also output (to the terminal) the frequency, background, and weight matrices for your given gapped n-mer and TF.

2 Required items

To run the scaled version of MARZ on HB, one will need the following things. (These are all included in the folder by default).

(a) A file of aligned sequences, here titled "Hb.txt". This is a text file consisting of newline-delimited aligned sequences, all of the same length. This is a text file that can be easily converted from a .fasta file by removing the first line.

(b) A collection of ChIP peaks, here contained in the folder "Hb". These must all be 100bp to fully match what was described in the paper, though certainly different lengths can be used (provided that the length of the smallest ChIP peak is larger than the length of the aligned sequences). All of the ChIP peak files must consist of a single line of text containing the appropriate nucleotides. These files must be named $0, 1, 2 \ldots, N-1$, (with no file extension) where N is the number of ChIP peaks, as is done in the folder Hb.

(c) A file containing background sequence, here the entire *Drosophila melanogaster* genome is contained in the file "entirechrom." Due to the large size of this file, it is not contained within the current folder but can be downloaded, as a zipped file, from the following website:

`https://www.amherst.edu/people/facstaff/jdresch/background-sequence-for-marz`

This file requires that subsequent chromosomes be separated by an empty line, so as to ensure that no set of nucleotides that would otherwise fall on two different chromosomes is scored. Additionally, this file *can* contain 'N's, and these will not be scored in the calculation of the background matrices.

Also helpful is a shell script for automating the running of MARZ and printing the terminal output to a file. This is provided as well, under the name "runsimplemarz.sh"

3 Deciding how to run the program

One example run of the program, contained in "runsimplemarz.sh", is the following:

```
./marz Hb.txt entirechrom 3142 Hb 4 0.5 hbtesting;
```

The first four parameters passed in should probably not be changed. They reflect the file of aligned sequences, the background genome, the number of ChIP peaks (N), and the folder of where to find those ChIP peaks, respectively.

The last three parameters represent the type id of the matrix to be tested, the threshold position to use, and the filename to save the results to, respectively. The type ID will be an integer corresponding to the gapped n-mer desired. Here, the type ID is 4, corresponding to $mkkm$. (For a full list of which type IDs correspond to which gapped n-mers for $n \leq 6$, see Table 1 in the paper).

The threshold position to use ought not be confused with the actual threshold T to use. Though both are in the range $[0, 1]$, recall that a threshold position of .5 will in this case correspond to the threshold T such that the 50% lowest scoring aligned sequences will not be considered binding. So, when I ran the above command, I got that $T = 0.936331$.

4 Making sense of the output

The output will be a tab delimited text file (in the above example, saved as "hbtesting") containing a table such as the following:

Type	TypeName	Threshold	Score	Hits	Borderlines	FalsePositives	False Negatives
4	mkkm	0.936331	0.563495	427	2687	249.74	2695

The results are fairly straightforward. Type corresponds to the type ID of the gapped n-mer, and the type name corresponds to its string representation. Threshold corresponds to the threshold T that was generated, and Score refers to the RZ score that was calculated.

The Hits column corresponds to the number of ChIP peaks that the matrix was able to successfully tell apart. The Borderlines column corresponds to the number of ChIP peaks that the matrix was not able to tell apart from its set of scrambled representations. To find the number of misses, one must take the number of ChIP peaks N and subtract the number of hits and borderlines. Here, the number of misses is

$$3192 - 427 - 2687 = 78.$$

Last, the false positives and false negatives column correspond to the number of false positives and false negatives, whose definition is in the paper.

5 Actually running the program

Open up the terminal (here using the example of bash) and change directory to the directory holding the code. Then, type "make" to compile the code. Last, run

```
bash runsimplemarz.sh
```

where this file should contain your (customized if desired) parameters to input to the Marz algorithm. Expect to see results in about 5 minutes.
